# Short Report: Lack of Diurnal Variation in Salivary Cortisol Is Linked to Sleep Disturbances and Heightened Anxiety in Adolescents with Williams Syndrome

**DOI:** 10.3390/bs13030220

**Published:** 2023-03-03

**Authors:** Jessica Hayton, Atiqah Azhari, Gianluca Esposito, Ray Iles, Michaella Chadiarakos, Giulio Gabrieli, Dagmara Dimitriou, Stephen Mangar

**Affiliations:** 1 Sleep Education and Research Laboratory, UCL Institute of Education, 25 Woburn Square, London WC1H 0AA, UK; 2 Psychology and Human Development, UCL Institute of Education, London WC1H 0AA, UK; 3 Psychology Programme, School of Humanities and Behavioural Sciences, Singapore University of Social Sciences, Singapore 599494, Singapore; 4 Affiliative Behaviour and Physiology Lab, Department of Psychology and Cognitive Science, University of Trento, 38068 Rovereto, Italy; 5 Department of Veterinary Medicine, University of Cambridge, Cambridge CB2 1TN, UK; 6 Neuroscience and Behaviour Laboratory, Italian Institute of Technology, 00161 Roma, Italy; 7 Department of Clinical Oncology, Imperial College Healthcare NHS Trust, Charing Cross Hospital, London W6 8RF, UK

**Keywords:** Williams Syndrome, cortisol, anxiety, sleep, adolescents

## Abstract

Objective: The aim of the current study was to examine the potential relationship between sleep patterns, cortisol levels, and anxiety profiles in adolescents with Williams Syndrome (WS) compared to typically developing adolescents. Method: Thirteen adolescents with WS and thirteen TD adolescents (age range 12–18 years) were recruited. Participants were provided with a “testing kit”, containing instructions for collecting data through a sleep diary, MotionWare actigraphy, the Childhood Sleep Habits Questionnaire (CSHQ), and the Spence Children’s Anxiety Scale, and a salivary cortisol collection kit. Results: Adolescents in the WS group did not show diurnal variation in salivary cortisol. Significantly higher scores were reported for two CSHQ subsections, night wakings and parasomnias, in the WS group. Regarding the actigraphy, only significantly longer sleep latency was observed in the WS group. In comparison to the TD group, the WS group had significantly higher anxiety. As expected, the TD group showed typical diurnal variation in cortisol, whereas the WS group showed a flattened cortisol profile throughout the day. Conclusions: From the developmental perspective, this study provides new data supporting the conclusion that sleep problems are not transient but continue to persist into adolescence in WS. Future studies ought to consider examining the role of cortisol and its interplay with anxiety levels and sleep problems across the lifespan in individuals with WS.

## 1. Introduction

Sleep disturbances are commonly reported in children with developmental disorders. Poor sleep habits may trigger impairments in cognitive and behavioural functioning [1,2], including lower academic performance [3], reduced attentional capacities (e.g., [4]), and poor executive functioning [5]. A suboptimal quality of sleep has also been associated with adverse and challenging anti-social behaviours [6], such as aggression, tantrums, non-compliance, and impulsivity [2,7,8]. Sleep disturbances have been found to be predictors of a reduced quality of life, related to stress, depression, and overall family functioning [9,10]. In childhood, a reduced sleep duration has been associated with developmental delays in language acquisition and consolidation [11,12]. Sleep disturbances have also been reported in infants with neurodevelopmental disabilities such as Williams Syndrome [13], which raises the importance of early identification and intervention during development, as these could mitigate against the cognitive and behavioural consequences of poor sleep.

Williams Syndrome (WS) is a rare neurodevelopmental disorder affecting approximately 1 in 20,000 individuals in the United Kingdom. WS is equally prevalent in male and female populations, and is not specific to ethnicity [14]. The disorder is caused by a micro-deletion of approximately 26–28 genes from the long arm of chromosome 7 at the point q11.23 [14]. Sleep concerns have been reported in infants with WS [15]. In early childhood, children with WS have been found to display long sleep latencies, reduced sleep efficiency and increased night wakings [16,17,18,19], frequent bed wetting and sleep anxiety [16,17], and movements during sleep [20]. Moreover, a few studies have reported an atypical sleep architecture, comprised of reduced rapid eye movement (REM) and amplified slow-wave sleep (SWS) stages [21,22]. Additionally, associations between elevated bedtime cortisol, a sleep-related hormone, and sleep onset have been reported in children with WS [23].

Cortisol has an established 24-h circadian rhythm with peak levels in the morning, particularly up to around thirty minutes after waking, before its concentration decreases throughout the day, with the lowest level being observed before night-time [24,25]. It has been argued that there is a bi-directional relationship between sleep deprivation and the secretion of cortisol, where poorer sleep is often associated with higher cortisol levels [24,26,27]. However, several recent studies have shown a rather different pattern. For instance, [28] observed a flattened curve in individuals with sickle-cell disease, which was associated with deficits in neurocognitive profiles that were moderated by sleep quality [28]. In another study, mothers of autistic children who exhibited a lack of diurnal variation suffered from sleep disturbances and high depression scores [9].

The present study aimed to examine the relationships between sleep patterns, anxiety, and cortisol levels in adolescents with WS compared to TD healthy adolescents. We hypothesised that (1) adolescents with WS would show a different cortisol profile than the age-matched TD controls, and (2) the cortisol profiles would be related to sleep parameters and anxiety scores.

## 2. Materials and Methods

### 2.1. Participants

Thirteen adolescents with WS were recruited via the database of the Williams Syndrome Foundation, UK. The control group included typically developing (TD) adolescents who were matched by chronological age to the WS cohort (see Table 1). None of the TD participants were diagnosed with any learning disability, nor had they received treatment for sleeping problems at the time of testing. English was used as a first language for all recruited participants. Ethical approval was granted from the Institute of Education, UCL Ethics Committee (grant number: 72838) and the Williams Syndrome Foundation, UK. Written informed consent was obtained from all parent/guardians, and consent was also secured from each child participant.

### 2.2. Measures

#### 2.2.1. Child Sleep Habits Questionnaire (CSHQ)

The CSHQ [29] is a standardised 45-item parent report measure for assessing sleep habits and potential sleep difficulties in children.

#### 2.2.2. Spence Children’s Anxiety Scale (SCAS)

The Anxiety Scale [30] more broadly measures the severity of the anxiety symptoms that are outlined in DSM-IV, including panic and aggravation, separation anxiety, obsessive–compulsive disorder, and generalised anxiety. The medical history questionnaire gathered data on other conditions that may have an impact on sleep, e.g., asthma, health habits, and diet.

#### 2.2.3. Actigraphy

The MotionWatch8, manufactured by CamNtech, is a CE-marked Class 1 medical device with FDA approval (K132764). The actiwatch was worn on the non-dominant wrist for 5 weekday nights within the same week. The actigraphy collected movement samples in 30-s epochs. The following measures were recorded: bedtime, get-up time, time in bed, assumed sleep, actual sleep (duration), actual sleep (%), sleep efficiency (%), sleep latency, and the fragmentation index. A sleep diary was used in conjunction with actigraphy to record the time children went to bed.

#### 2.2.4. Salivary Cortisol

Mothers were asked to take three samples of their children’s saliva on one weekday of the week during which they wore the actigraphy watch. An oral fluid collector (OFC) consisting of a synthetic polymer swab designed to collect 0.5 mL of saliva mixed with 3 mL of OFC buffer was used to collect the samples (Soma Bioscience Ltd., Wallingford, UK). With this technique, samples are stable at room temperature for several weeks and are unaffected by recent food and drink ingestion. Salivary cortisol was determined using an enzyme immunoassay (EIA) test kit (Soma Bioscience Ltd., Wallingford, UK) and read by an automated analyser (Tecan Nanoquant, Männedorf, Switzerland). The assay range for cortisol was 0.25–32.0 ng/mL. In the case where maximum values were obtained, samples were titrated and re-analysed. Cortisol profiles were assessed using all 3 values to determine if each participant had a flat or normal cortisol profile compared to the individual group mean.

Each participant was required to provide salivary samples for 3 time points over a 24-h period. The timings were: (a) afternoon at approximately 4 p.m. (baseline); (b) 30 min before habitual bedtime; (c) 30 min after waking. Either parents or participants themselves (under adult supervision) were asked to place a cotton/polymer swab in their mouth for 30–45 s in order to collect each sample.

### 2.3. Analytical Plan

Data were analysed using Python (v. 3.11.1, Centrum voor Wiskunde en Informatica (CWI), Amsterdam, The Netherlands). Independent samples *t*-tests were conducted to examine the results of the CSHQ, SCAS, and cortisol secretion. Mann–Whitney U tests were employed to compare actigraphy measures by group. Finally, to examine the relationship between cortisol levels, sleep habits, and anxiety scores, a two-way multivariate analysis of variance (MANOVA) was conducted.

## 3. Results

### 3.1. Child Sleep Habits Questionnaire

Table 2 shows the results from the indepedent *t*-test. Eighteen participants had a cut-off score that was greater than the clinical threshold of ≥41, of whom nine were from the TD group and nine were from the WS group. There was no outlier present in the CSHQ data.

### 3.2. Spence Children’s Anxiety Scale

Table 3 shows the results from the independent *t*-test analysis of differences in anxiety measures between the TD and WS groups. Statistically significant differences were found in four out of six SCAS subscales (panic and aggravation, separation, obsessive compulsive, and generalised anxiety) and the total SCAS score. No statistical difference was found between groups in the subscales of physical injury and social anxiety. There were no outliers observed for the SCAS measures.

### 3.3. Actigraphy

Table 4 depicts the results for the independent samples *t*-tests comparing actigraphy (supported by the sleep diary) and sleep variables between the WS and TD groups. The sleep diary data were used to support bed- and get-up-time data and showed that adolescents with WS had earlier bed- and get-up-times compared to TD adolescents.

It should be noted that the sleep data from objective actigraphy were not in line with the data reported from the CSHQ. The actigraphy data showed that the WS group had a greater sleep latency than the TD group, but this difference between groups was not reported on the CSHQ sleep onset delay subscale. However, it is important to note that the CSHQ provided additional information, namely, a higher number of night wakings and parasomnias in the WS group.

### 3.4. Salivary Cortisol

Table 5 shows the means and standard deviations for both groups. Salivary cortisol samples were collected from all 26 participants, though only 22 participants (n = 13 WS; n = 9 TD) provided sufficient samples for quantification. Only one morning sample for a participant with WS did not contain enough to be quantified. Three samples for one TD participant were also removed due to the participant’s incorrect storage of the buffer (returned upside down), which led to elevated and spurious findings.

**Figure 1:**
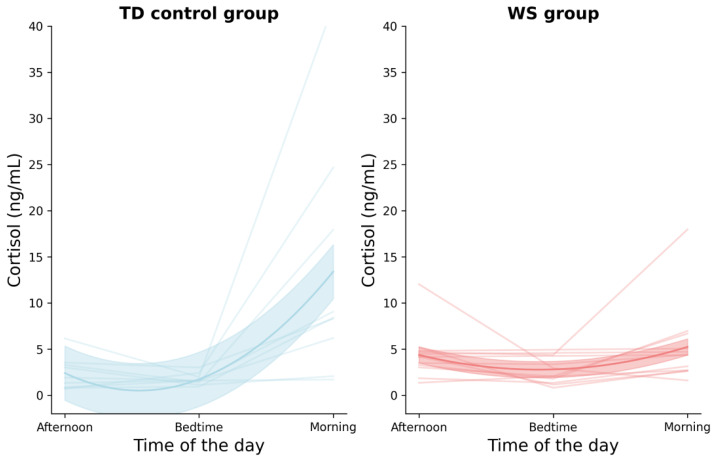
Plot depicting cortisol concentration for TD and WS groups across three time points: baseline (afternoon), bedtime, and morning. Note: each line in the graphs represents data from a unique participant.

No statistical significance between cortisol levels was found in the WS sample. Statistically significant differences were found in the TD samples between bedtime and morning samples and between baseline and morning samples Table 6. Figure 1 illustrates the cortisol concentrations of the TD and WS groups for three data points: the baseline, bedtime, and morning time points. In the TD group, the morning cortisol level was significantly higher than both the baseline and bedtime time points. In contrast, in the WS group, the cortisol levels remained relatively unchanged across all three time points.

### 3.5. Sleep, Anxiety, and Cortisol

To investigate the association between cortisol profiles, sleep quality, and anxiety scores, a multivariate analysis of variance (MANOVA) was conducted, with the cortisol levels at the three different time points as the dependent variables and the CSHQ and SCAS scores as the independent variables. The results, shown in Table 7, revealed no significant or interaction effects between sleep habits or anxiety and cortisol profiles.

## 4. Discussion

Adolescence is considered a sensitive period, during which some of the largest sleep disruptions occur (e.g., [31,32]). This “perfect storm” has been related to pubertal changes, alterations in the circadian rhythm (including later bed- and wake-times), and changes in social functioning [33]. The current study supported the notion that problematic sleep is a general characteristic of adolescence, as most participants (82%, n = 11 TD; n = 10 WS) were categorised as having sleep disturbances. However, unlike TD adolescents, who restricted their sleep duration, the range of sleep problems was varied and persistent across developmental stages in individuals with WS. Actigraphy data showed that adolescents with WS spent a longer time in bed and experienced more sleep, albeit marginally, compared to their TD peers. The National Sleep Foundation recommendations suggest that adolescents should optimally receive 8–10 h of sleep per night [34]. However, the actual mean duration of sleep was 7:36 for the WS group and 7:05 for the TD group.

The sleep profiles obtained from the CSHQ and actigraphy suggested that both groups experienced disturbed sleep. Similar to previous studies, the actigraphy data and the parental report (CSHQ) indicated discordance (see [17,35]). The findings and effect sizes of the CSHQ and actigraphy data suggested that it is suitable for the two measures to be used in conjunction with each other in young populations. Used together, the instruments can provide more holistic information on different aspects of sleep health profiles, which could be informative for healthcare professionals in the sleep management of patients.

Previous work has indicated that sleep-related problems in adolescence are closely associated with elevated anxiety levels [36], and this relationship was explored in the context of cortisol in the current study. Compared to the TD group, the WS group showed a lack of diurnal cortisol variation, with similar concentrations observed across the three time points. This pattern was in line with recent studies (e.g., [28]), which similarly revealed that individuals with sickle-cell disease showed a flattened diurnal cortisol curve. It is important to note that the literature currently lacks a sufficient understanding of the optimal cortisol secretion within this developmental period. To date, researchers are still not cognizant of how the different medical conditions in individuals with WS might impact cortisol secretion and interact with the sleep/wake cycle.

Significant results were reported in the WS group relative to the total anxiety score, including the subscales of general anxiety, separation anxiety, and panic and aggravation. These findings suggested that adolescents with WS experienced higher total anxiety compared to their TD peers, which could explain the larger effect sizes in the sleep anxiety dimension of the CSHQ. This result concurred with the established literature, according to which elevated general anxiety is considered a persistent behavioural phenotype in WS [37,38,39,40], as is separation anxiety [41] and panic-related anxiety [42,43,44]. Non-significant findings were reported in the following anxiety subscales: physical injury, obsessive–compulsive disorder, and social anxiety. Arguably, this could be due to the pro-social behaviour exhibited by adolescents with WS. Further, the effect sizes indicated trends in terms of variance, which suggested that a larger sample size could have potentially yielded a statistically significant result. Though the prevalence of anxiety in individuals with WS exceeded that in TD individuals, its persistence over time is relatively stable compared to TD adolescents (who have been noted to experience remission within the first 12 months of diagnosis [40,45]). Chronic anxiety in individuals with WS might be associated with the lack of variation in cortisol secretion, though caution needs to be exercised here, as this finding ought to be examined further.

The findings presented here were based on a cross-sectional design with a small sample size of participants at the adolescent stage of life only. In the continued search for the possible mechanisms involved in the interaction between sleep, anxiety, and cortisol, future studies should employ either a longitudinal approach to examine individuals with WS across the lifespan, or adopt a larger collaborative international study that could buttress the sample size. Finally, future research could further examine the interplay and associations between cortisol, actigraphy, sleep disturbances, and anxiety in WS populations.

## Figures and Tables

**Table 1:** Characteristics of the groups.

Characteristic	Williams Syndrome	Typically Developing	*t* Stat.	$\chi^{2}$	*p*-Value
N	13	13			
Sex	6 Females, 7 Males	8 Females, 5 Males	.	0.619	0.734
Age (months)	180.77 ± 24.67	180.69 ± 18.15	0.009	.	0.993

**Table 2:** Group mean scores, standard deviations, and group differences in CSHQ.

Subscale	WS	TD	*t* Stat.	*p*-Value	Cohen’s d
Bedtime Resistance	7.08 (2.47)	6.69 (1.18)	0.51	0.617	0.20
Sleep Onset Delay	2.23 (0.93)	1.77 (0.83)	1.34	0.194	0.52
Sleep Duration	4.92 (2.10)	4.46 (1.71)	0.61	0.545	0.24
Sleep Anxiety	5.23 (1.69)	4.46 (0.66)	1.53	0.140	0.60
Night Wakings	4.92 (2.25)	3.54 (0.78)	2.09	0.047 *	0.82
Parasomnias	9.54 (2.33)	7.77 (1.42)	2.34	0.028 *	0.92
Sleep Disordered Breathing	3.85 (1.68)	3.31 (0.63)	1.08	0.289	0.43
Daytime Sleepiness	12.69 (3.77)	13.54 (2.44)	−0.68	0.503	-0.27
**Total**	48.15 (8.51)	43.54 (4.68)	1.71	0.100	0.67

* *p* < 0.05.

**Table 3:** Group mean scores, standard deviations, and group differences in SCAS.

SCAS Subscale	WS	TD	*t* Stat.	*p*-Value	Cohen’s d
Panic nand Aggravation	4.23 (3.90)	0.92 (1.89)	2.75	0.011 *	1.08
Separation	5.08 (3.75)	1.69 (1.65)	2.98	0.007 **	1.17
Physical Injury	4.77 (3.30)	3.38 (2.33)	1.24	0.228	0.49
Social	4.46 (2.82)	6.38 (4.15)	−1.38	0.180	−0.54
Obsessive Compulsive	3.38 (3.48)	0.69 (1.44)	2.58	0.016 *	1.01
Generalised	6.23 (3.00)	2.08 (1.50)	4.46	<0.001 ***	1.75
Total	28.15 (14.55)	14.69 (9.73)	2.77	0.011 *	1.09

* *p* < 0.05, ** *p* < 0.01, *** *p* < 0.001.

**Table 4:** Group mean scores, standard deviations, and group differences in actigraphy.

Actigraphy	WS	TD	U	*p*-Value	Cohen’s d
Bedtime	21:25 (0:53)	22:34 (1:01)	34.0	0.010 *	1.16
Get-up Time	07:26 (0:56)	08:19 (1:07)	41.5	0.029 *	0.81
Time in Bed	09:55 (0:54)	09:06 (1:07)	57.0	0.104	0.72
Assumed Sleep	08:56 (0:54)	08:21 (0:48)	49.0	0.375	0.65
Actual Sleep	07:36 (0:48)	07:05 (0:43)	48.5	0.389	0.65
Sleep Latency	01:10 (0:49)	0:41 (0:38)	60.0	0.057	0.63
Fragmentation Index	30.05 (3.35)	28.1 (6.11)	51.0	0.285	0.35
Actual Sleep	85.28% (2.9)	84.91% (3.72)	31.0	0.536	0.10
Sleep Efficiency	76.92% (5.14)	78.31% (6.55)	31.0	0.536	0.22

* *p* < 0.05.

**Table 5:** Means (and standard deviations) of cortisol concentration at baseline (afternoon), bedtime, and morning for WS and TD groups.

Time	Cortisol (nMol/L)		*t* Stat.	*p*-Value	Cohen’s d
	WS	WD			
Afternoon	4.23 (2.61)	2.40 (1.79)	1.83	0.082	0.79
Bedtime	2.76 (1.41)	1.73 (0.65)	2.04	0.054	0.89
Morning	5.24 (4.33)	13.39 (13.06)	−2.03	0.056	0.90

**Table 6:** Comparison by group of cortisol concentrations at baseline (afternoon), bedtime, and morning.

Group	Comparison	*t* Stat.	*p*-Value	Cohen’s d
WS	Afternoon vs. Bedtime	1.79	0.087	0.70
	Afternoon vs. Morning	0.70	0.487	0.28
	Morning vs. Afternoon	1.95	0.063	0.78
TD	Afternoon vs. Bedtime	1.05	0.310	0.49
	Afternoon vs. Morning	2.50	0.024 *	1.18
	Morning vs. Afternoon	2.68	0.017 *	1.26

* *p* < 0.05.

**Table 7:** Results of MANOVA analysis on cortisol levels, sleep quality and anxiety socres.

Effect	λ	df_1_	df_2_	F Stat.	*p*-Value
CSHQ Score	0.9465	3	15	0.28	0.837
SCAS Score	0.9560	3	15	0.23	0.873
CSHQ × SCAS	0.9534	3	15	0.24	0.864

λ = Wilks’ lambda.

## Data Availability

Not applicable.
